# Biodegradation Assessment of Poly (Lactic Acid) Filled with Functionalized Titania Nanoparticles (PLA/TiO_2_) under Compost Conditions

**DOI:** 10.1186/s11671-019-2891-4

**Published:** 2019-02-14

**Authors:** Yanbing Luo, Zicong Lin, Gang Guo

**Affiliations:** 1 School of History and Culture, National Center for Experimental Archaeology Education, Sichuan University, Chengdu, 610064 China; 2 State Key Laboratory of Biotherapy and Cancer Center, West China Hospital, West China Medical School, Sichuan University, Chengdu, 610065 China

**Keywords:** Biodegradation, PLA, Functionalized TiO_2_, Compost

## Abstract

This paper presents a biodegradation study conducted for 90 days under standardized controlled composting conditions of poly (lactic acid) (PLA) filled with functionalized anatase-titania nanofiller (PLA/TiO_2_ nanocomposites). The surface morphology, thermal properties, percentage of biodegradation, and molecular weight changes at different incubation times were evaluated via visual inspection, scanning electron microscopy (SEM), X-ray diffraction (XRD), differential scanning calorimetry (DSC), and gel permeation chromatography (GPC) by taking degraded samples from compost at the end of target biodegradation time interval. The rapid increase of crystallinity indicated that the PLA and PLA/TiO_2_ nanocomposites had heterogeneous degradation mechanisms under controlled composting conditions. The biodegradation rate of PLA/TiO_2_ nanocomposites was higher than that of pure PLA because water molecules easily penetrated the nanocomposites. The dispersion of the nanoparticles in the PLA/TiO_2_ nanocomposites affected the biodegradation rate of PLA. Moreover, the biodegradation of PLA could be controlled by adding an amount of dispersed TiO_2_ nanofillers under controlled composting conditions.

## Introduction

Poly (lactic acid) (PLA), a synthetic biodegradable polymer, is investigated worldwide for biomedical and consumer applications because of the increasing need for renewable materials that are sustainable alternatives to petrochemical-derived products [1–4]. PLA is the product that results from the polymerization of lactide or lactic acid, which is the most extensively produced carboxylic acid in nature by microbial fermentation of carbohydrates [5]. However, the applicability of PLA has been relatively limited because its heat distortion temperature, toughness, and degradation rate are unsatisfactory [6, 7]. One of the methods to resolve these drawbacks is to modify PLA by adding inorganic nanoparticles, including typical nanoclay, carbon nanotubes, zinc oxide, and anatase (A-TiO_2_) [8–15]. Recently, the PLA/TiO_2_ nanocomposites were prepared by us via melting blending PLA with chemically modified TiO_2_ (solution lactic acid grafted TiO_2_, hereafter referred to as g-TiO_2_) [16]. Results showed that TiO_2_ nanoparticles had a significant effect on the improvement of the mechanical properties of the PLA/TiO_2_ blends, such as strain at break and elasticity, compared with pure PLA. At the same time, g-TiO_2_ nanoparticles had a strong influence on hydrolytic degradation and photodegradation of PLA [17, 18].

The study of biodegradability and biodegradation mechanism of biodegradable materials using laboratory-scale test is an extremely important method from industrial and scientific point of view which provides understanding of the service life of these materials [15]. There are several methods currently available to assess the biodegradability of biodegradable materials, which are in general based on an indirect measurement, such as carbon dioxide production, biogas generation, or oxygen consumption [19, 20].

Biodegradation characteristics of PLA in compost have been studied and reported [21–23]. Composting is an accelerated biodegradation of organic materials in a warm, moist, and aerobic environment under a combination of microbial population and controlled composting conditions [24, 25]. Moreover, the biodegradation of PLA in composting conditions, a temperature- and humidity-dependent process, involves several processes, namely, water uptake, ester cleavage, and formation and dissolution of oligomer fragments [26]. The most accepted mechanism of the PLA biodegradation involves a two-step degradation process. Initially, the heat and moisture in the compost attack the PLA chains and split them apart, thereby producing small Mw polymers and, eventually, lactic acid. Thereafter, the microorganisms in the compost and soil mineralize the oligomer fragments and lactic acid to generate methane and carbon dioxide (CO_2_) under anaerobic and aerobic conditions, respectively [27–29].

Recently, the effect of fillers on the biodegradation of PLA has attracted great attention and particular attention has been focused on nanofillers, such as nanoclays, carbon nanotubes, and hydroxyapatite [23, 30–38]. Some authors [32–34] found out that adding nanoparticles could accelerate biodegradation of PLA, which was attributed to the high relative hydrophilicity of the nanoparticles, thereby enabling the easy permeability of water into the polymer matrix and triggering hydrolytic degradation. However, other studies [35–38] reported that biodegradation was retarded because of the enhanced barrier properties of the nanocomposites.

Although there have been some literatures focusing on the biodegradation of PLA materials, the role that TiO_2_ plays in PLA degradation remains controversial. How did the TiO_2_ nanoparticles affect the biodegradation of PLA was not clear. So, a study of the biodegradation of PLA, modified by TiO_2_ nanofillers under compost condition, is still needed. The current study, based on the estimation of the evolving CO_2_, assessed the biodegradation of PLA/TiO_2_ nanocomposites extensively under controlled laboratory compost conditions, a complement of degradability of the PLA/TiO_2_ nanocomposites under different degradation conditions, could extend PLA’s use in various end-use applications in the future.

## Methods

### Materials

PLA (manufactured by Natureworks^@^ (4032D)) exhibited a weight-average molecular weight (Mw) of 19,600 kDa and polydispersity of 1.89 as determined through gel permeation chromatography (GPC). PLA dried at 65 °C for 24 h under reduced pressure and stored in vacuum with humidity absorber before use. Lactic acid (88%, Guangshui National Chemical Co.) was distilled at 80 °C to remove water before use. The anatase titania nanoparticles, with an average primary particle size of ca. 20 nm, were supplied by Pangang Co., Ltd. Toluene and chloroform were used as received. Chromatographic grade microcrystalline cellulose was supplied by Shanghai Chemical Reagent Co., Ltd. The composting inoculums, which were obtained from an organic fraction of municipal solid waste (MSW), were supplied by the Degradable Plastics Professional Committee of the China Plastics Processing Industry Association (CPPIA).

### Sample Preparation

Detailed information on the functionalization of the TiO_2_ nanoparticles and preparation of the PLA/TiO_2_ nanocomposites has been reported [16]. G-TiO_2_ nanofillers were prepared by grafting lactic acid oligomer onto anatase surface. PLA/TiO_2_ nanocomposites were prepared by melt blending via a corotating twin-screw extruder. Pure PLA was subjected to same mixing treatment so as to have the same thermal history as nanocomposites. The samples with 0, 0.5, 1.0, 2.0, 5.0, 8.0, and 15.0 wt% g-TiO_2_ were prepared and labeled as PLA, PLA/TiO_2_–0.5, PLA/TiO_2_–1, PLA/TiO_2_–2, PLA/TiO_2_–5, PLA/TiO_2_–8, and PLA/TiO_2_–15 nanocomposites.

Small chip specimens of PLA and g-TiO_2_ at different ratios were converted into sheets of approximately 0.5 mm in thickness by pressing at 190 °C for 4 min under 10 MPa followed by cooling at room temperature for 5 min under 5 MPa. Thereafter, the compression molded samples were cut into 5 mm × 5 mm size and weighed.

### Degradation Tests

A biodegradation test was conducted in a laboratory scale-installation based on standard test methods designed for biodegradable plastics (GB/T19277–2003/ISO 14855-1:2005) (determination of the ultimate aerobic biodegradability of plastic materials under controlled composting conditions—method by analysis of evolved CO_2_). Most of the carbon in the metabolized substrates generates energy through chemical transformation to CO_2_ in aerobic environments [39]. Therefore, measurements of the generation of CO_2_ are considered the most appropriate measure of biodegradation in most circumstances. The standard specifies a procedure to determine the ultimate aerobic biodegradability by measuring the amount of evolved CO_2_ and percentage of the biodegradation degree of the test materials under controlled composting conditions. The composting inoculum was obtained from an organic fraction of MSW, which was sieved to sizes under 5 mm. Thereafter, the fine fraction was used as inoculums. Table 1 shows the determined physicochemical properties of the composting inoculums. In each test, a series of composting reactors (each sample in triplicates) was loaded with 15 g of the reference material (i.e., microcrystalline cellulose (MCE), which was suggested by the standard) or test material (each film weighted and labeled before degradation), 85 g of inoculum, and 320 g of dry sea sand (provides good homogeneous conditions and an improved aerobic environment inside the inoculum). Thereafter, the reactors were placed in an incubator without light at 58 ± 2 °C through an experiment time of 90 days. Aeration was initiated using water-saturated CO_2_-free air; the flow rate through each reactor was set at 25 mL·min^−1^. The humidity, mixing, and aeration in all the reactors were controlled as established by the GB/T19277–2003/ISO 14855-12,005 requirements. At selected times, three to four specimens of each sample were selected, washed with distilled water, and dried at room temperature at least 24 h to constant weight.

**Table 1:** Physicochemical properties of inoculums

Properties	Value	Test method
pH	7.2	GB/T 19277–2003 ISO 14855-2005
Total dry solid (TS) %	71.3	
Volatile solids (VS, % on TS)	19.3	
Residual ash content (RAC, % on TS)	80.7	
Moisture^a^ (%)	53.5	
C/N ratio	15	

^a^Percentages are expressed on a dry weight loss

The CO_2_ that evolved during the biodegradation process was trapped in NaOH solutions and measured at regular intervals using titration method. The NaOH was titrated with standard HCl solution to the phenolphthalein endpoint. The total CO_2_ evolved during biodegradation was calculated with reference to the control flask. The data reported for each sample was the mean value obtained from three samples.

### Characterization

#### Microscope Examination

Scanning electron microscopy (SEM) images were obtained using a Philips FEI INSPECT F instrument operated at 5 kV. All specimens were sputter coated with gold prior to analysis.

#### Thermal Analysis

Thermal properties of samples were studied by differential scanning calorimetry (DSC) (TA Q20, TA Instruments). Thermograms were obtained under nitrogen flow (50 mL/min) at a heating and cooling rates of 10 °C/min in the temperature range from room temperature to 200 °C and from 200 to − 50 °C, respectively.

#### XRD Studies

X-ray diffraction (XRD) analyses were performed using a DX-1000 X-ray diffractometer (Dandong Fanyuan Instrument Co. LTD. China) equipped with a Cu K_*α*_ (*λ* = 0.154 nm) source. The generator was operated at 25 mA and 40 kV. Samples were scanned at different angles (i.e., from 2 to 70°) at a scanning rate of 6°/min.

#### Determination of the Percent of Biodegradation (*D*_*t*_, %)

Percent of biodegradation (*D*_*t*_, %) could be calculated using Eq. 1, which was adopted as for Eq. 2 [1, 40].

1 $$ {D}_t\left(\%\right)=\frac{{\left({\mathrm{CO}}_2\right)}_T-{\left({\mathrm{CO}}_2\right)}_B}{{\mathrm{Th}}_{\mathrm{CO}2}}\times 100 $$

where (CO_2_)_*T*_ is the amount of CO_2_ (in g/flask) evolved from the test materials, (CO_2_)_*B*_ is the amount of CO_2_ (in g/flask) evolved in the control flask, and Th_CO2_ is the theoretical CO_2_ amount produced by the polymeric materials.

The theoretical CO_2_ amount that can be produced in each flask (Th_CO2_, g^2^/g sample) was calculated using the following equation:

2 $$ {\mathrm{Th}}_{\mathrm{CO}2}={M}_{\mathrm{TOT}}\times {C}_{\mathrm{TOT}}\times \frac{44}{12} $$

where *M*_TOT_ is the total weight (g) of the dry polymeric solids material added into the composting flask at the start of the experiment, *C*_TOT_ is the weight (g) of the total organic carbon in the total dry polymeric solids in the sample, and 44 and 12 are the molecular mass of CO_2_ and atomic mass of *C*, respectively.

### Molecular Weight Measurement

The molecular weights of the PLA nanocomposites before and after composting were determined through GPC. The GPC system was equipped with a Waters 1515 Isocratic HPLC pump, a Waters 2414 refractive index detector, and Waters 717 plus autosampler. Chloroform was used as eluent at 0.8 mL/min flow rate at 30 °C. Calibration was accomplished with polystyrene standards.

## Results and Discussion

**Fig. 1:**
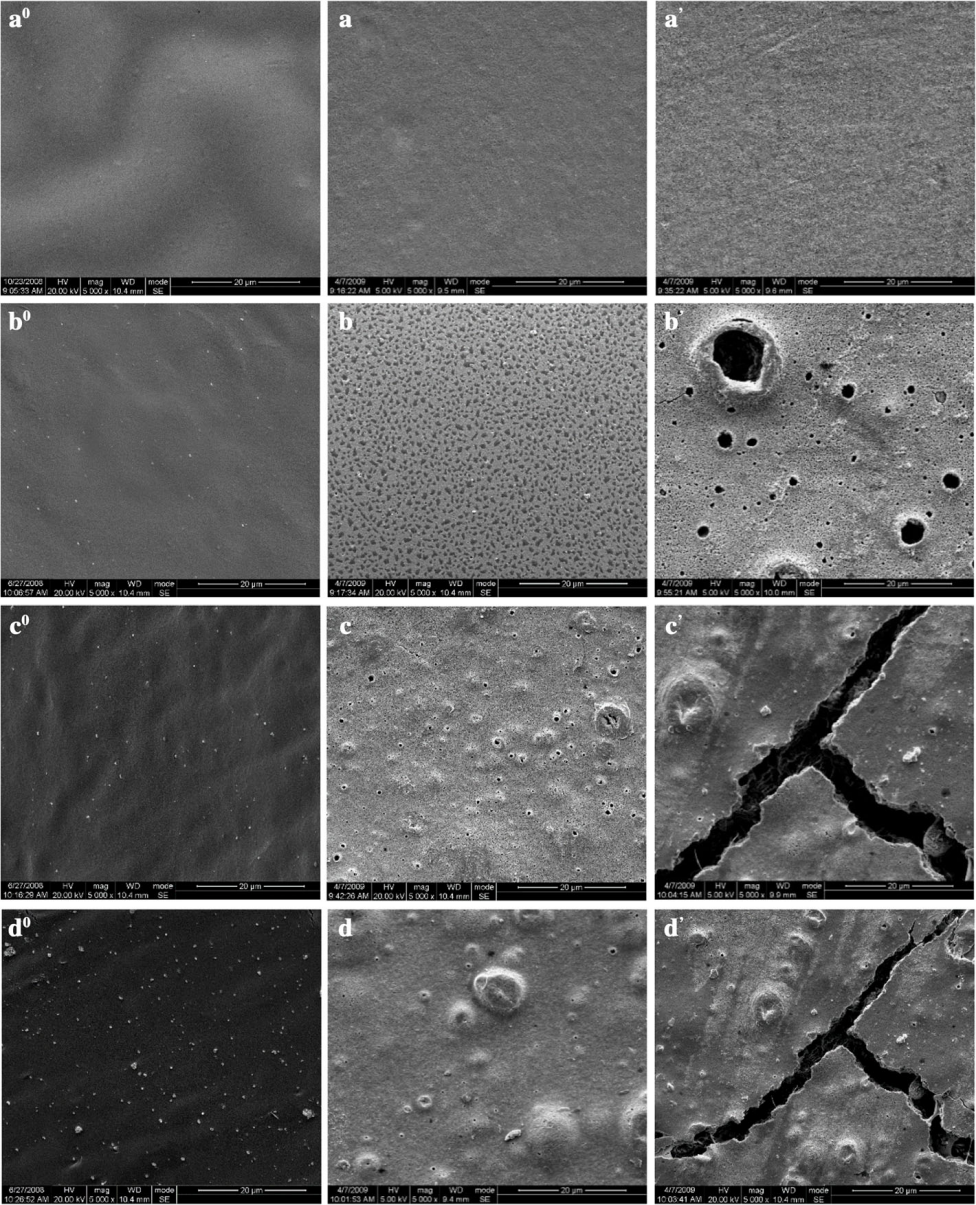
SEM photography of the surface of pure PLA (**a**^**0**^, **a**, **a’**), PLA/TiO_2_–2 (**b**^**0**^, **b**, **b’**), PLA/TiO_2_–5 (**c**^**0**^, **c**, **c’**) and PLA/TiO_2_–8 (**d**^**0**^, **d**, **d’**) nanocomposites as a function of incubation time. **a**^**0**^, **b**^**0**^, **c**^**0**^, **d**^**0**^:0 day; **a**, **b**, **c**, **d**: 5 days; **a’**, **b’**, **c’**, **d’**: 20 days

Polymer degradation is associated with changes in characteristics, such as color, surface morphology, and mechanical properties. The temporal changes in the appearance of the pure PLA and PLA/TiO_2_ nanocomposites were different under laboratory conditions. The pure PLA matrix surfaces, which were initially transparent in agreement with the amorphous structure, became relatively whitish after 2 days of biodegradation [41]. This feature increased with incubation time until complete opacity after 10 days. Yellowy to dark brown plaques, caused by water permeation and microorganisms incubation, began to be observed on the surface of neat PLA films surface after 30 days. However, large area of dark brown plaques emerged on the surface of PLA nanocomposite after 6 days (figure was not shown). The brown spots imply the microorganism colonies and the cracks represent the biodegradation effect. Figure 1 shows the surface morphology of PLA and its TiO_2_ nanocomposites under SEM observation. Before degradation, the surface of neat and PLA/TiO_2_ nanocomposites were smooth. Neat PLA did not present significant changes on the surface after biodegradation 5 days in compost conditions. After 20 days, the surface roughness of neat PLA increased (Fig. 1a, a’). However, PLA/TiO_2_ nanocomposite exhibited progressive changes clearly showing that considerable degradation of PLA/TiO_2_ composite occurred. Obvious cracks and voids (Fig. 1b, b’; c, c’; and d, d’; respectively) were observed on the surface of nanocomposites. This could be owned to the hydrolysis of PLA and microorganisms activities. With increasing incubation time, the cracks and voids became substantially deep and large (Fig. 1 b’, c’, and d’, respectively), thereby suggesting chain loss and surface erosion as time progresses. The bulk erosion phenomenon for all test materials was similar to the hydrolytic degradation process of the PLA and PLA/TiO_2_ nanocomposites [17].

**Fig. 2:**
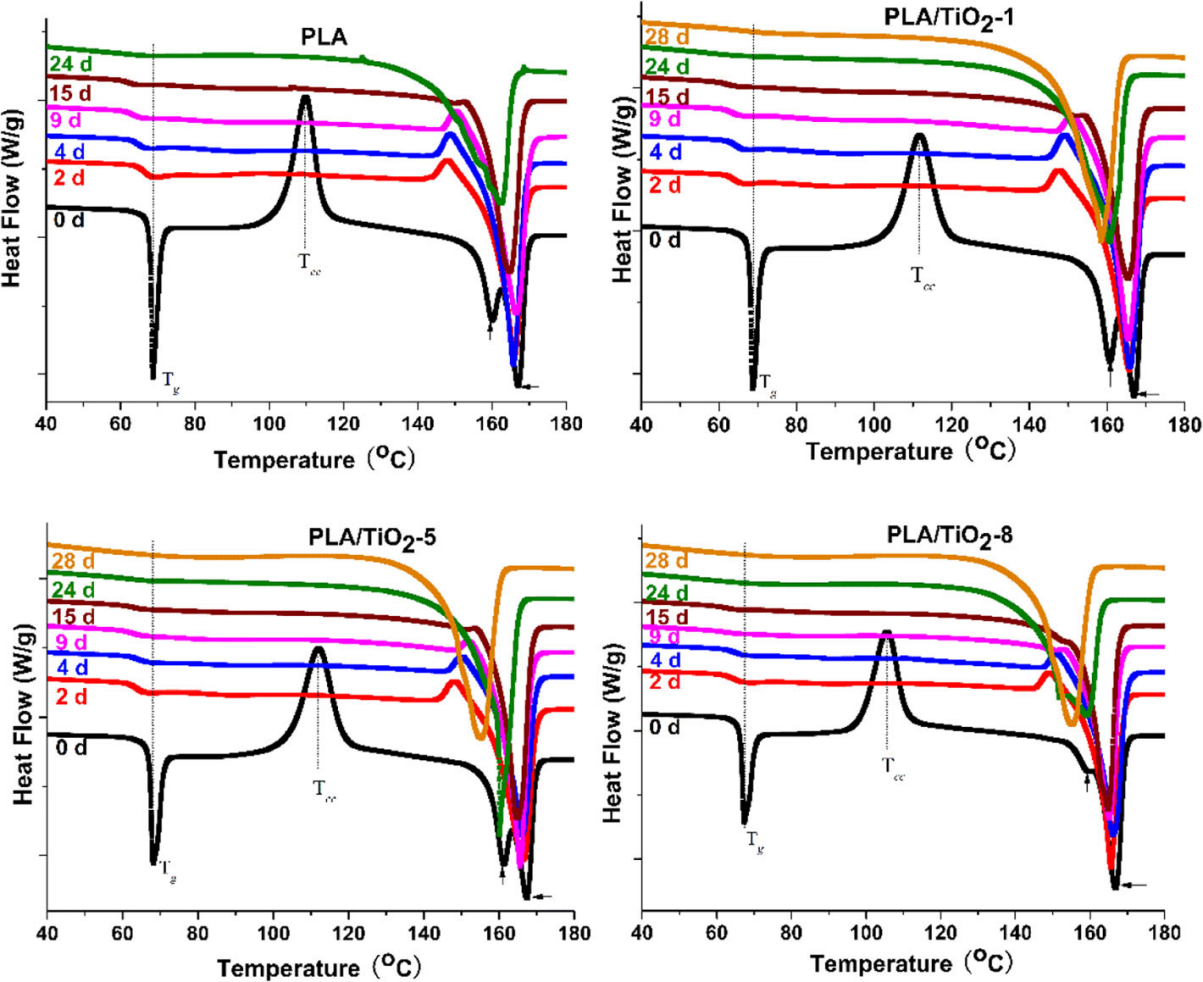
DSC thermograms of biodegradation products of pure PLA and PLA/TiO_2_ nanocomposites at different incubation times, first heating scan

**Fig. 3:**
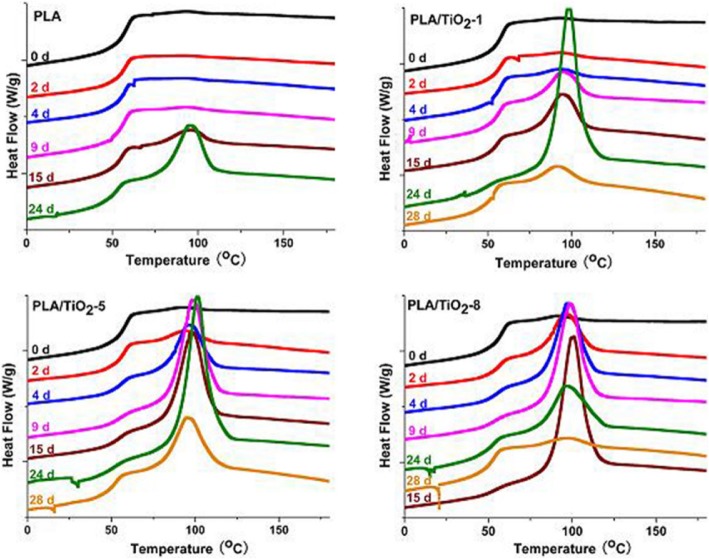
DSC thermograms of biodegraded pure PLA and PLA/TiO_2_ nanocomposites at different incubation times, cooling scan

To evaluate the crystallinity of the PLA and PLA/TiO_2_ nanocomposites during biodegradation, samples selected at different incubation times were analyzed for their thermal properties (Figs. 2 and 3). Figure 2 shows that the glass transition temperature (*T*_*g*_) decreased slightly for all samples with degradation time. The decrease of *T*_*g*_ was clearly due to an enhanced mobility of the molecules, as a consequence of the hydrolysis process and plasticizing effect of the oligomer fragments and water during biodegradation [33, 42]. The disappearance of cold crystallization peak (*T*_*cc*_) for all of the samples only after 2 days could be ascribed to the hydrolysis of PLA and the rapid increase in crystallinity of the polymer matrix [43]. The decrease of *T*_*m*_ was ascribed to the rapid molecular mass reduction [44, 45]. The bimodal melting peak gradually changed to monomodal peak, thereby implying that the small and imperfect crystals disappeared with degradation time. This result proved that the degradation of PLA proceeded rapidly in the amorphous regions during the early stage of degradation under controlled composting conditions. The cooling scan (see Fig. 3) shows that the crystallization peak of the neat PLA increased gradually. However, the crystallization peaks of the PLA/TiO_2_ nanocomposites initially increased significantly and decreased slightly thereafter with an increase in incubation time. Moreover, the higher the nanofillers content was, the earlier the crystallization peaks arrived at its peak. The decrease of crystallization peak further verified that the crystalline region began to degrade after the degradation of the amorphous region. Giuliana and Roberto [42] reported that at short times for PLA sample some amorphous regions change into crystal, then the crystallinity degree increases due to the effect of the erosion of the amorphous parts. Moreover, the crystalline regions undergo hydrolysis at long times.

**Fig. 4:**
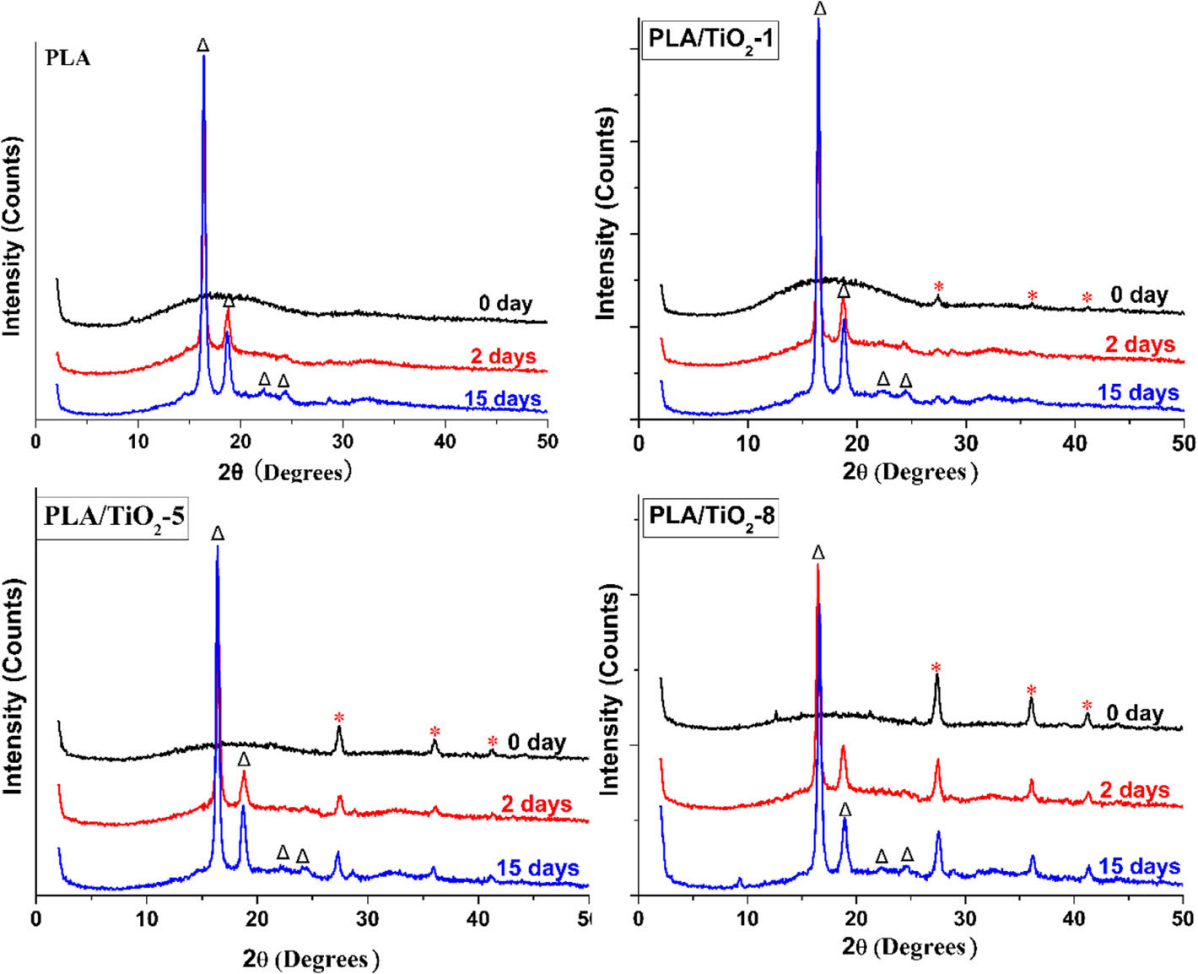
XRD patterns of pure PLA and PLA/TiO_2_ nanocomposites at different incubation times

XRD provides an ideal method to monitor changes in the crystallization of polymers during degradation. The XRD patterns of PLA and its nanocomposites (Fig. 4) show that the polymer matrix maintains an amorphous structure before biodegradation. Only after 2 days, two strong peaks at 2*θ* = 16.4, 18.5°, 20.9°, and 23.6° clearly appeared and their intensity increased with incubation time. This result implied that poly (L-lactide) or poly (D-lactide)-type crystalline structures were formed [46, 47]. The change of crystalline peak indicated that amorphous regions degraded more rapidly than crystalline regions, which increased the crystalline-to-amorphous regions ratio value. This result was consistent with DSC results and the transparency change of the samples.

**Fig. 5:**
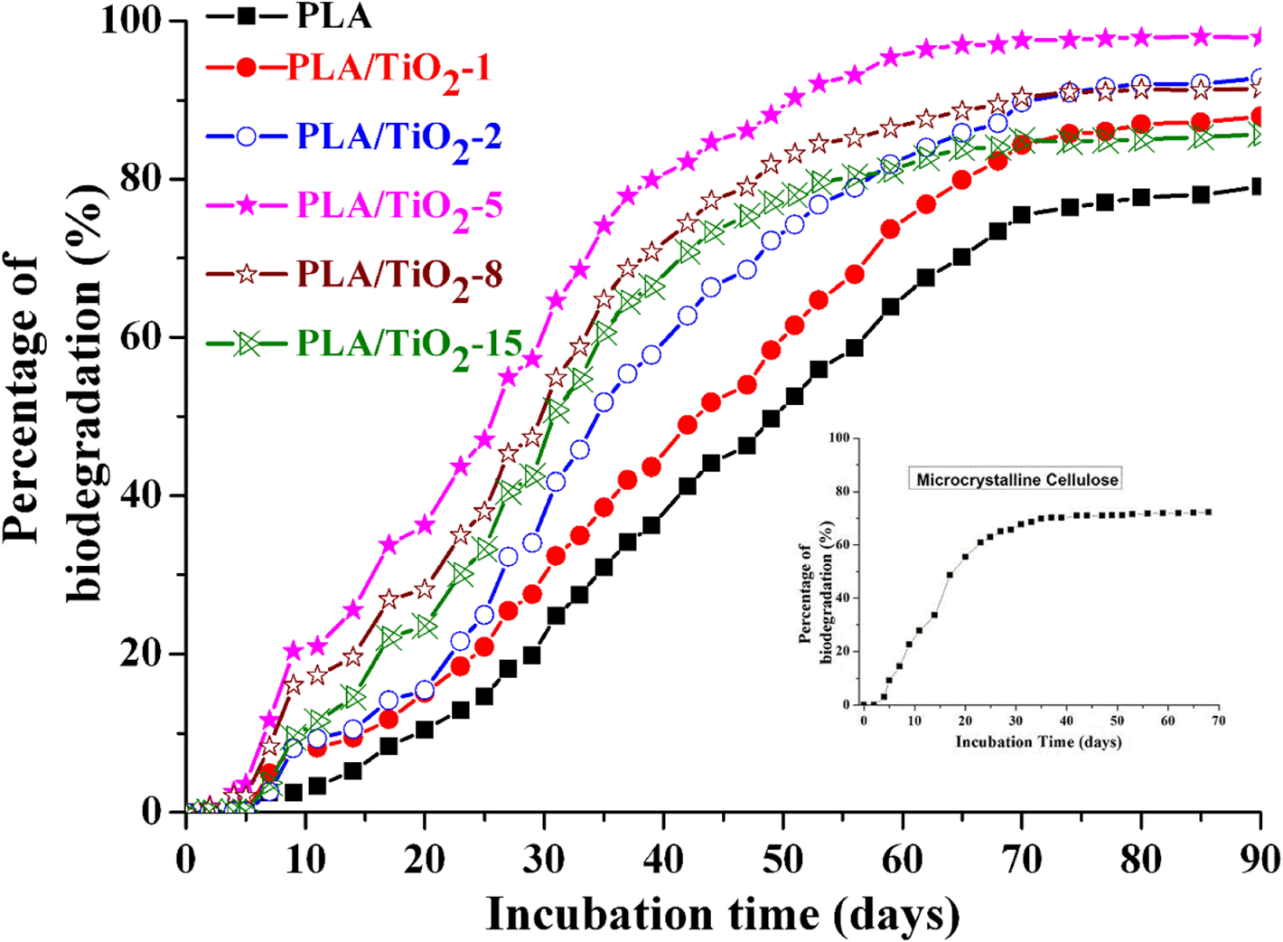
Percentage of biodegradation as a function of incubation time for pure PLA and PLA/TiO_2_ nanocomposites. The insert is the percentage of biodegradation as a function of time for microcrystalline cellulose

The evaluation of the inoculum validation is crucial during biodegradation under composting conditions. The activity of the inoculum was measured as required by the standard method: *D*_*t*_ of the reference material should be at least 70% at the end of the 45 days of testing. The insert in Fig. 5 shows that MCE begins to biodegrade after 5 days, and the percentage of biodegradation is up to 72% at the end of the 45 days of incubation. These result indicated that MCE in the experiment was effective as a reference material. In the experiment, duplicate composting vessels showed good reproducibility (standard deviation ± 1.3%). Figure 5 shows *D*_*t*_ for neat PLA and PLA/TiO_2_ nanocomposites during incubation. A similar behavior was observed for the PLA and PLA/TiO_2_ nanocomposites, that is, a lag phase was first observed which is then followed by a steep linear increase in biodegradation and thereafter by a plateau phase for all samples. The steepness of the increase should be indicative of increased degradation. However, the curves indicated that the lag phase of the nanocomposites was a little shorter than that of pure PLA. This result indicated the presence of TiO_2_, at some degree, accelerated the initial phase of degradation and increased the percentage of CO_2_ produced at the end of the incubation period. After 80 days of incubation under controlled composting conditions, *D*_*t*_ for PLA, PLA/TiO_2_–1, PLA/TiO_2_–2, PLA/TiO_2_–5, PLA/TiO_2_–8, and PLA/TiO_2_–15 reached up to 78.9, 86.9, 92.0, 97.8, 91.3, and 85.0%, respectively. Kunioka et al. [48] reported that the final biodegradability of PLA was 80%. The results from our experiment showed that *D*_*t*_ of the commercially pure PLA product was also nearly 80% at the end of 80 days. The decrease of *D*_*t*_ beginning from PLA/TiO_2_–8 is ascribed to the intense agglomeration of TiO_2_ when its content was beyond 8 wt% [16]. Further details are presented in the following section.

**Fig. 6:**
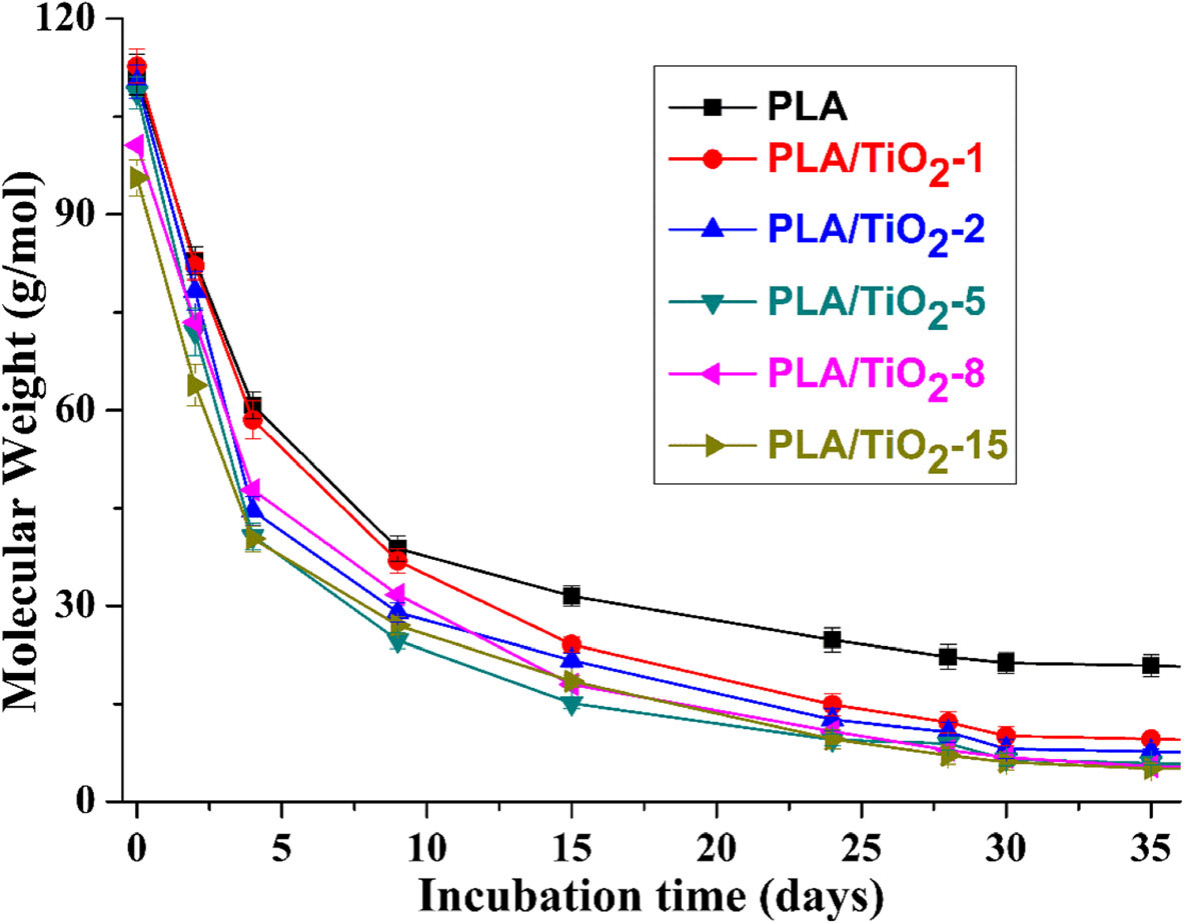
Change of Mn as a function of time for pure PLA and PLA/TiO_2_ nanocomposites

The different percentages of biodegradation are related to the different molecular weight changes of the polymer matrix. Figure 6 shows the molecular weight change of the samples as a function of incubation time. The curves show that the changes of Mn in the PLA/TiO_2_ nanocomposites were similar (i.e., a rapid decrease of Mn followed by a plateau phase of nearly a constant Mn) at least in the determined incubation time. To explore the degradation mechanisms caused by the addition of nanofillers, a model that accounts for autocatalysis by the generated carboxylic acid end groups was used to calculate the catalyzed degradation rate constant according to reference [17, 49]:

3 $$ \ln {M}_{nt}=\ln {M}_{n0}- kt $$

where *k* is catalyzed hydrolytic degradation rate constant, *M*_n0_ is the number-average molecular weight before degradation, *M*_*nt*_ is the number-average molecular weight at any time.

**Fig. 7:**
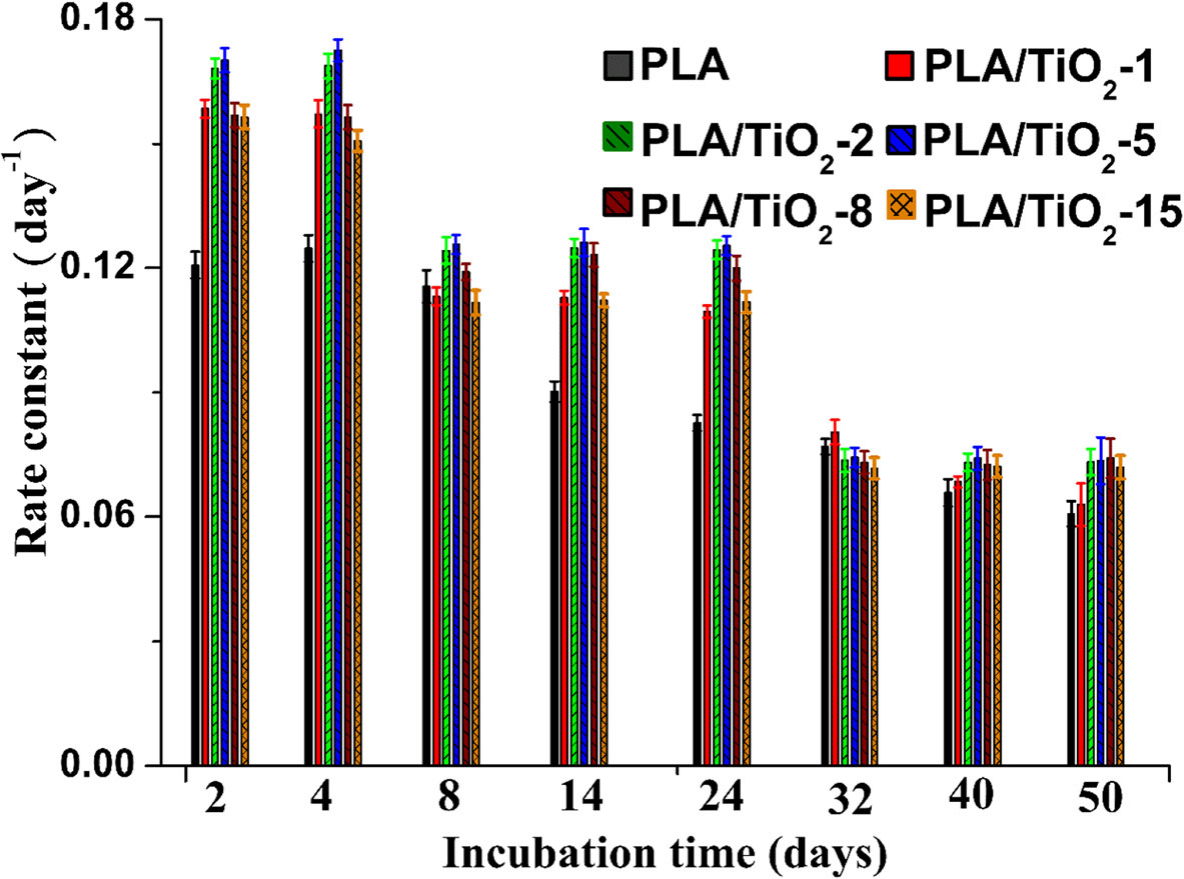
Biodegradation rate versus incubation time for pure PLA and PLA/TiO_2_ nanocomposites

The *k* values evaluated by Eq. (3) are plotted in Fig. 7. From Fig. 7, the degradation rate of the PLA and PLA nanocomposites could be identified to two and three phases, respectively. Mn decreased rapidly during the first 8 days and followed by a plateau phase thereafter for neat PLA. For PLA/TiO_2_ nanocomposites, the highest values of *k* means that *M*_*n*_ decreased rapidly in the first phase (i.e., from 0 to 4 days). The following 5 to 24 days are ascribed to the second phase, and the values of *k* decreased slightly compared with the first phase. Few studies [13, 50] showed that the crystalline part of PLA was more resistant to degradation than the amorphous part; thus, the decrease in *k* in this phase could be caused by the increase of crystallinity of the PLA matrix. After 24 days (i.e., the last phase), the value of *k* decreased again. At this stage, the polymer completely degraded into oligomer fragments or lactic acid, and the microorganisms mineralized the remaining materials to continuously generate CO_2_.

Under composting conditions, the factors that affect the biodegradation tendency of PLA would control the degradation of the PLA/TiO_2_ nanocomposites. When an amount of g-TiO_2_ was homogeneously dispersed in the PLA matrix, the water molecules penetrated easily within the samples to trigger the degradation process [17]. Consequently, Mn decreased substantially in the first phase. The evolution of the lag phase of CO_2_ for PLA and its nanocomposites during this period indicated that microorganisms need suitable polymer chains to mineralize. With increased incubation time, the polymer chains in amorphous regions degraded and the number of amorphous regions decreased; thus, the percentage of crystalline to the amorphous region (i.e., χ_c_) increased [39], thereby leading to the decrease of *k* in the second phase. However, the oligomer fragments began to be mineralized by microorganisms in this stage, thereby indicating that the productive phase for the PLA mineralization occurred. With the decrease of the remaining oligomer fragments and the increase of χ_c_, *k* and *D*_*t*_ decreased and a nearly long plateau phase was observed for *k* and *D*_*t*_ in the third stage. In our previous study [16], the morphology of each nanocomposite was reported and determined through SEM and TEM; the results showed that the dispersion of g-TiO_2_ with under 5 wt% in the PLA/TiO_2_ nanocomposites was better than that obtained with a high concentration of nanofillers. In terms of the dispersion and content of TiO_2_, PLA/TiO_2_–5 had the largest *k* and *D*_*t*_ compared with the other nanocomposites in our experiment.

## Conclusions

PLA/TiO_2_ nanocomposites were prepared (based on PLA and functionalized g-TiO_2_) and subjected to biodegradation under controlled composting conditions. Using such a standard, the information of patterns on the surface of the samples and the rapid increase of crystallinity indicated that the PLA and PLA/TiO_2_ nanocomposites had heterogeneous biodegradation mechanisms. The degradation study of nanocomposites under composting conditions showed that the inherent degradable character of PLA remained after the incorporation of functionalized titania nanoparticles (PLA/TiO_2_). The addition of the TiO_2_ nanoparticles increased the degradation rate of the PLA matrix because the water molecules easily penetrated the PLA/TiO_2_ nanocomposites, thereby activating the degradation process. This phenomenon was particularly evident for PLA/TiO_2_–5 because of its high TiO_2_ content and good dispersion of TiO_2_ nanofillers in the PLA matrix compared with other nanocomposites.

## Abbreviations

DSC: Differential scanning calorimetry
*D*_*t*_: Percent of biodegradation
GPC: Gel permeation chromatography
g-TiO_2_: Grafted TiO_2_
MCE: Microcrystalline cellulose
Mn: Number-average molecular weight
Mw: Weight-average molecular weight
PLA: Poly (lactic acid)
SEM: Scanning electron microscopy
T_*cc*_: Cold crystallization peak
T_*g*_: Glass transition temperature
XRD: X-ray diffraction
